# Knockdown of Telethonin Reduces Contractions and Provokes Aberrant Ca^2+^-waves in Human iPS Cell-induced Cardiomyocytes

**DOI:** 10.14789/ejmj.JMJ24-0025-OA

**Published:** 2025-06-20

**Authors:** TETSUYA HANDOH, TOMOHIKO AI, HARUKA INABA, HAYATE YAMAMOTO, YUNA HORIUCHI, ATSUSHI HORI, ISIK TURKER, YOKO TABE, TAKASHI MIIDA

**Affiliations:** 1 Department of Clinical Laboratory Medicine, Juntendo University Graduate School of Medicine, Tokyo, Japan; 1 Department of Clinical Laboratory Medicine, Juntendo University Graduate School of Medicine, Tokyo, Japan; 2 Department of Clinical Laboratory Medicine, Juntendo University School of Medicine, Urayasu Hospital, Chiba, Japan; 2 Department of Clinical Laboratory Medicine, Juntendo University School of Medicine, Urayasu Hospital, Chiba, Japan; 3 Department of Clinical Laboratory Technology, Faculty of Medical Science, Juntendo University, Chiba, Japan; 3 Department of Clinical Laboratory Technology, Faculty of Medical Science, Juntendo University, Chiba, Japan; 4 Department of Cardiology, Washington University School of Medicine, St. Louis, Missouri, USA; 4 Department of Cardiology, Washington University School of Medicine, St. Louis, Missouri, USA

**Keywords:** telethonin, *TCAP*, iPS cell, cardiomyopathy, arrhythmias

## Abstract

**Objectives:**

Dilated cardiomyopathy (DCM) is one of the leading causes of heart failure. To date, 48 genes are known to be associated with DCM. Telethonin, encoded by the *TCAP* gene, is a Z-disk protein that composes cytoskeletal structures and facilitates various signaling pathways in cardiomyocytes. At least, six *TCAP* variants have been found in patients with DCM. We sought to investigate the role of *TCAP* in cardiac function using *TCAP*-knockdown (KD) iPS cell (iPSC)-induced cardiomyocytes (CMs).

**Methods & Results:**

To investigate the role of *TCAP* in cardiomyocytes, the *TCAP* gene was knocked down in human iPS cells established from a healthy subject (201B7) using the CRISPR-Cas9 genome editing. The expressions of *TCAP* mRNA and telethonin were confirmed by RT-qPCRs and Western blot, respectively. The 201B7 wild type (WT) and the *TCAP*-knocked down (KD) cells were differentiated into cardiomyocytes (CMs). The contractility measured by a high-resolution block matching-based optical flow technique showed that all contractility parameters, including contraction velocity, relaxation velocity, and contraction-relaxation duration, were decreased in the KD-CMs (n = 54) compared to the WT-CMs (n = 24) (WT vs. KD: 51.28 ± 22.82 vs. 29.11 ± 22.83 µm/s, *p* < 0.001; 22.05 ± 8.85 vs. 12.48 ± 8.85 µm/s, *p* < 0.001; 0.82 ± 0.12 vs. 0.58 ± 0.12 s, *p* < 0.001). Ca^2+^-imaging studies showed aberrant Ca^2+^-waves in the KD-CMs.

**Conclusions:**

We found that *TCAP*-KD in human iPS cell-induced CMs impairs contraction, induces triggered activities, and abnormal Ca^2+^-waves, which is consistent with the phenotypes of DCM.

## Introduction

Dilated cardiomyopathy (DCM) is one of the leading causes of congestive heart failure (CHF)^1)^. It has been reported that cardiomyopathies can be caused by genetic variants. To date, 48 genes are known to be associated with DCM^2)^.

Telethonin or TCAP, encoded by the gene *TCAP*, is an important member of the Z-disk proteins, and *TCAP* variants have been reported to be associated with DCM^3)^. Since Hayashi et al. reported a *TCAP* variant in a DCM patient in 2004^4)^, only six heterozygous missense variants have been detected in DCM patients^5-8)^. Since functional study has not been performed, the genetic evidence is insufficient.

Experimental evidence is also quite limited. Impaired expression of *TCAP* in mice hearts can disrupt the Z-disk structures and cause abnormal cardiac Ca^2+^-handling under stress^9, 10)^. In theory, the *TCAP*'s association with cardiomyopathy can be supported by protein-protein interactions with other DCM-associated proteins such as titin (TTN)^3, 9, 11)^. Currently, due to insufficient genetic and experimental evidence, its gene-disease association is classified as “limited” by ClinGen (https://search.clinicalgenome.org/kb/affiliate/10008?page=1&size=25&search=).

To further investigate the role of *TCAP* in human hearts, we knocked down the *TCAP* using a gene editing method in human iPS cells (iPSCs). After confirmation of knockdown (KD) of the protein by Western blotting, we measured the contractile motion properties of the iPSC-derived cardiomyocytes after knockdown of *TCAP* (KD-CMs) using a motion field imaging, a high-resolution block matching-based optical flow technique^12)^, and compared it to the contraction of the wild type (WT) iPSC-induced cardiomyocytes (WT-CMs). Ca^2+^-handling was also measured using a Ca^2+^- imaging. Our data revealed that the contraction was impaired and aberrant abnormal Ca^2+^-waves appeared in the KD-CMs compared to the WT- CMs. Although we do not know the details of the underlying mechanisms yet, our human iPSC-derived cardiomyocyte model recapitulated the phenotypes of cardiomyopathy.

## Materials and Methods

### Generation and maintenance of iPSCs

In this study, we used the 201B7 iPSC line generated from a healthy individual. The iPSCs were cultured using a feeder-free method and maintained in 6-well plates coated with 0.5 mg/mL iMatrix-511 silk (892021, Matrixome, Osaka, Japan) in StemFit^®^ AK02N (RCAK02N, AJINOMOTO, Tokyo, Japan) at 37°C and 5% CO_2_. The medium was replaced every other day, and the iPSCs were passaged at a density of 0.3 × 10^6^ cells per 6-well plate for each passaging.

### iPSC cardiac differentiation

The iPSCs were differentiated into cardiomyocytes (iPS-CMs) with Cardiomyocyte Differentiation Kit (A2921201, Thermo Fisher Science, Waltham, MA, USA), which includes Cardiomyocyte Differentiation Medium A, B and Cardiomyocyte Maintenance Medium. Three mediums were replaced every other day. The iPS-CMs were maintained in 12-well plates coated with 2% Geltrex™ LDEV-Free, hESC-Qualified, and Reduces Growth Factor Basement Membrane Matrix (A1413302, Thermo Fisher Science, Waltham, MA, USA) at 37℃ and 5% CO_2_.

### CRISPR-Cas9 gene editing

*TCAP* was knocked down using CRISPR-Cas9 gene editing techniques. We designed a synthetic CRISPR RNA (crRNA; Integrated DNA Technologies (IDT), Skokie, IL, USA) to target the most upstream Exon (i.e., Exon 1; Please see Supporting information). Since *TCAP* consists of only two exons, Alt-R™ CRISPR HDR Design Tool (IDT) showed only a few candidates of guide RNA in Exon 1. A ribonucleoprotein (RNP) complex was created by combining the Alt-R Cas9 Nuclease 3NLS and the crRNA: tracrRNA duplex (1072532, IDT, Skokie, IL, USA). The RNP complex was introduced into 1.0 × 10^6^ iPSCs by electroporation using the NEPA Porator (Nepa Gene, Chiba, Japan). After electroporation, cells were cultured for 1 week and then dissociated into single cells using Accumax (#07921, STEMCELL TECHNOLOGIES, Vancouver, Canada). Single colonies were picked and screened by sanger sequencing to confirm the sequence of the targeted site. Two gene-corrected isogenic control iPSC clones were generated and employed in this study.

### Real-time quantitative reverse transcription PCR

The expression of *TCAP* mRNA was verified using real-time quantitative reverse transcription polymerase chain reaction (PCR). Total RNA was extracted from iPSC-CMs with the RNeasy Plus Mini Kit (74134, QIAGEN, Hilden, Germany) and quantified using a NanoDrop 2000 Spectrophotometer (ND-2000, Thermo Fisher Scientific, Waltham, MA, USA). The RNA was then transcribed into complementary DNA (cDNA) using SuperScript™ IV VILO™ Master Mix (11756050, Thermo Fisher Scientific, Waltham, MA, USA). Quantitative PCR (qPCR) was conducted with TaqMan™ Fast Advanced Master Mix (4444556, Thermo Fisher Scientific, Waltham, MA, USA). Gene expression levels were normalized to the *GAPDH*, and the comparative change was evaluated using the threshold cycle (ΔCT) method described elsewhere^13)^.

### Western blotting

The expression of telethonin was confirmed using Western blotting. Total protein was extracted from iPSC-CMs using Cell Lysis Buffer (10X) (#9803, Cell Signaling Technology, Danvers, MA, USA), which was diluted to 1X and combined with cOmplete™ Mini Protease Inhibitor Cocktail (04693116001, Roche, Basel, Switzerland). The protein concentration was measured using Pierce™ BCA Protein Assay Kits (23227, Thermo Fisher Scientific, Waltham, MA, USA). Proteins were separated by standard SDS-PAGE and transferred to a PVDF membrane (IPVH00010, Merck, Darmstadt, Germany). The membranes were blocked with 1% skim milk and subsequently incubated with a 1 : 1,000-diluted anti-Telethonin antibody (ab121868, Abcam, Cambridge, UK) and a 1 : 4,000-diluted anti-mouse IgG, HRP-linked antibody (#7076, Cell Signaling Technology, Danvers, MA, USA). Chemiluminescence signals using Clarity Western ECL Substrate (1705060, Bio-Rad Laboratories, Hercules, CA, USA) were detected by the LAS4000 (GE Healthcare Japan, Tokyo, Japan). Protein expression levels were normalized to the β-Actin, and the comparative change between WT and KD was evaluated using ImageJ (http://imagej.nih.gov/ij/) (National Institutes of Health, Bethesda, MD, USA).

### Contractility measurement

The contractility was measured by a high-resolution block matching-based optical flow technique (SI-8000, SONY, Tokyo, Japan) as previously reported^14)^. A field stimulation was applied to the 21-day-old iPSC-CMs cultured in 12-well plates at 1 Hz with 5 ms depolarizing pulses at 50 V using platinum electrodes and an electronic stimulator (SEN-3401MG, Miyuki Giken, Tokyo, Japan).

### Ca^2+^-imaging

Intracellular Ca^2+^ levels were measured using Cal-520 (21130, AAT Bioquest, Inc., Sunnyvale, CA, USA) as previously described^15)^. Briefly, iPSC- derived cardiomyocytes cultured on polymer coverslip-bottom dishes were incubated with 4 μM Cal- 520/AM at 37°C with 5% CO_2_ for 30 minutes in Cardiomyocyte Maintenance Medium. The dishes were then washed with Tyrode buffer (in mM: 140 NaCl, 5.4 KCl, 5 HEPES, 1.2 MgCl_2_, 1.8 CaCl_2_, 10 glucose, pH 7.4). To prevent motion artifacts, 10 µM blebbistatin (027-17043, FUJIFILM Wako Chemicals, Osaka, Japan), an excitation-contraction uncoupler, was applied. The iPSC-CMs were stimulated at 1 Hz with 5 ms depolarizing pulses at 50 V using platinum electrodes. Data acquisition and analysis were conducted using AquaCosmos 2.0 (Hamamatsu Photonics, Hamamatsu, Japan). In this experiment, only the cells that were constantly captured by the field stimulation were evaluated. We defined ‘aberrant Ca^2+^-waves’ as irregular Ca^2+^-transients that were not respond to 1 Hz field stimulation.

### Data Analysis

Data were presented as median [interquartile range (IQR)], and comparisons were performed by nonparametric Mann-Whitney U test with p < 0.05 considered as statistically significant.

## Results

## The iPSC-derived cardiomyocytes

The iPSCs were differentiated into cardiomyocytes (iPS-CMs) as described in the Methods. Approximately after 14 days from start of differentiation, iPS-CMs began to beat spontaneously (data not shown).

## Knockdown of the *TCAP*

To investigate the effect of *TCAP* on cardiomyopathy, we knocked down *TCAP* from the WT- iPSCs using CRISPR-Cas9 gene editing techniques. The gene editing using the guide-RNA caused 14- nucleotide deletion (Figure S1 and S2), which might have caused frameshift (Figure S3) or splicing error (Figure S4 and S5).

**Figure 1:**
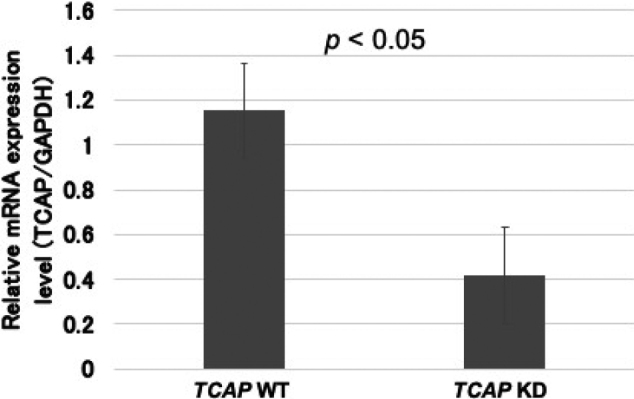
The expressions of *TCAP* mRNA measured by the RT-qPCRs Relative mRNA expression levels normalized by the *GAPDH* were decreased in *TCAP* KD (n = 5) compared to WT cells (n = 4).

**Figure 2:**
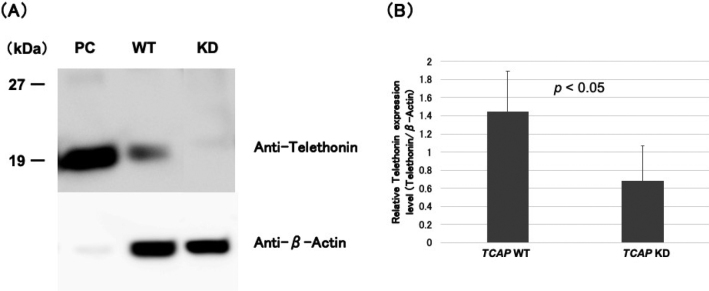
The expressions of telethonin measured by Western blotting (A) Representative data of Western blotting. Recombinant telethonin antigen was used as a positive control (PC). (B) Relative telethonin expressions normalized by the β-Actin were decreased to 47.1% in the *TCAP* KD (n = 5) compared to the WT cells (n = 5).

The gene expressions were analyzed with RT- qPCR. Figure 1 shows that *TCAP* mRNA levels normalized by the *GAPDH* were reduced to approximately 50% in the KD-CMs compared to the WT-CMs. In addition, the protein expressions were analyzed with Western blotting. Figure 2 shows that relative telethonin expressions normalized by the β-Actin were reduced to 47.1% in the KD-CMs compared to the WT-CMs (*p* < 0.05).

## Contractile functions

**Figure 3:**
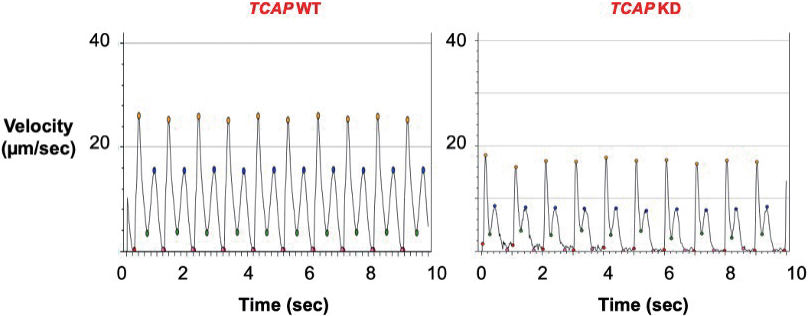
Representative motion vector waveforms obtained from contracting cardiomyocytes differentiated from *TCAP* WT and KD The vertical axis represents velocity (μm/s) and the horizontal axis represents time (s). The recording of the 10-s period of electrical stimulation (1-sec interval, 5 msec duration, 50 V) indicates that the biphasic waveforms represent myocardial contraction as the first peak (yellow plots), followed by relaxation as the second peak (blue plots).

**Figure 4:**
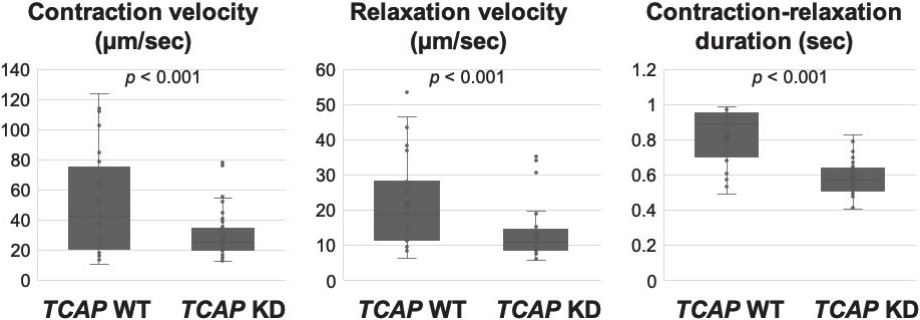
Summary data of contractility parameters All contractility parameters were decreased in the KD-CMs (n = 54) compared to the WT-CMs (n = 24).

Figure 3 shows representative motion vector waveforms obtained from contracting cardiomyocytes differentiated from *TCAP* WT and KD iPSCs. The recordings indicate biphasic waveforms consisting of contraction and relaxation components indicated as velocities. Compared to the WT-CMs, KD- CMs showed slower contraction and relaxation velocities. The contraction-relaxation duration was significantly shorter in KD-CMs than WT-CMs. Figure 4 shows a summary of contractility parameters.

## Ca^2+^-signaling

**Figure 5:**
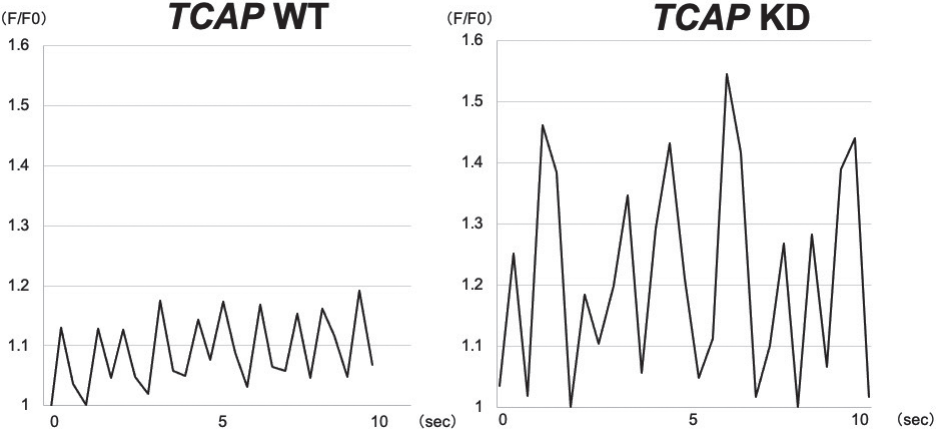
Representative trace of Ca^2+^-transients observed in iPSC-CMs The Ca^2+^-signals were recorded under 1 Hz field stimulation. Note that the aberrant Ca^2+^-waves persisted in the *TCAP*-KD CMs.

Figure 5 shows Ca^2+^-imaging obtained from the KD-CMs under pacing at 1 Hz with a field stimulation. While the WT-CMs rarely showed aberrant Ca^2+^-waves (n = 53), Ca^2+^-bursts were easily induced in the KD-CMs (n = 30) by the field stimulations, suggesting that the reduction of *TCAP* may increase arrythmia susceptibility.

## Discussion

Our study, for the first time, showed that knockdown of *TCAP* recapitulates the phenotypes of cardiomyopathies in human iPSC-induced cardiomyocytes.

Although *TCAP* variants, such as p.E13del, p.R18Q, p.E49K, p.R130C, and p.R158S, were found in DCM^6, 7, 8)^, the pathogenicity of these variants remains unknown. In the gnomAD (https://gnomad.broadinstitute.org/gene/ENSG00000173991?dataset=gnomad_r4), the minimal allelic frequencies (MAFs) of these variants are smaller than 0.001%, which is consistent with a rare monogenic disease according to the current standard operating procedure (SOP) for the gene curation by ClinGen (https://www.clinicalgenome.org/docs/variant-curation-standard-operating-procedure-version-3/). However, it is insufficient to define rare variant as causative only by MAFs^16)^. Another of the issue is that truncated variants have not been found in patients with DCM except in large chromosomal deletions (https://www.ncbi.nlm.nih.gov/clinvar/?term=cardiomyopathy) since null variants are usually thought to be pathogenic^17)^.

Though a *TCAP* knock out animal model could not recapitulate the phenotypes of human DCM^9)^, a biochemical study using yeast two-hybrid systems showed that *TCAP* can bind to the NH_2_ terminal (Z1-Z2) domains of titin (TTN) which is a main component for cardiac contractility^3)^. Also, overexpression of TTN Z1-Z2 disrupted the structures of Z-disk complex and α-actin filaments^3)^. Since telethonin is a key adaptor protein that also anchors actin, another important cytoskeletal component, to the Z-disk via MLP and α_2_-actinin^4)^, it is reasonable to speculate that telethonin plays an important role in cardiomyopathy as our data clearly showed that the cardiac contractile parameters were impaired by *TCAP*-KD.

As for our *TCAP*-KD model, the gene editing made a stop codon in Exon 2 (Figure S3 and S4), which can cause nonsense mediated decay (NMD). Though the stop codon in the last exon may not cause NMD, it is a theory and not necessary to occur. For example, Zhao did not detect TCAP protein in the zebrafish model in which homozygous knockout of *TCAP*^18)^. Their gene editing caused indel, leading to creation of stop codon in Exon 2. Thus, it is not necessary to see two bands in our heterozygous model.

Our data also demonstrated that *TCAP*-KD enhanced arrhythmogenesis (i.e., Ca^2+^-bursts) that is one of the important phenotypes of DCM. We are planning to investigate the effects of *TCAP*-KD on various ion channels since various cytoskeletal proteins including *TCAP* regulate ion channels^19-23)^.

## Limitations

There are several limitations in this study: (1) the iPSC-induced CMs are not matured as actual human cardiomyocytes; (2) we do not know the exact underlying mechanisms by which telethonin defects cause cardiomyopathy; (3) data were obtained by *in vitro* experiments, which is different from the actual human heart; (4) we don’t know the exact effects of *TCAP* KD on changes of cytoskeletal structures. We are planning to study the detailed structural changes using transmission electron microscopy. For example, if we observe disrupted Z-disk and disorganized A-bands, our model may recapitulate a DCM phenotype^24)^. If we observe myofibril disarray, it can be a hypertrophic cardiomyopathy (HCM) model though the *TCAP*'s association with HCM has been disputed by the ClinGen in 2022 (https://search.clinicalgenome.org/kb/genes/ HGNC:11610); (5) since we only evaluated the cells that were regularly stimulated, we do not know the contractile function of the cells that showed irregular Ca^2+^-waves; (6) since the KD cells were derived from a clone cell, they might have been more homogeneous than WT cells. This might have caused large variability in the contraction experiments (Figure 4); (7) The nucleotide deletion Figure S1 and S2) might have created a stop codon in Exon 2 (Figure S3). Alternatively, the SpliceAI simulation showed a high probability of loss of the splice donor site, which might have caused nonsense mediated decay (Figure S4 and S5). Though we do not know which was the real cause of the KD, our results showed reduced mRNA and proteins, indicating NMD took place. Also, this change took place in only one allele (Figure S2), which created a hetero- KD model for this study.

## Conclusion

In this study, we presented that knockdown of telethonin impaired contractile functions of iPSC- induced CMs compared to the WT-CMs. The KD- CMs also showed aberrant Ca^2+^-waves like triggered activities. These data indicate that telethonin is involved in the pathophysiological mechanisms of DCM. Currently, we are creating knock-in iPSC clones harboring *TCAP* pathogenic variants found in patients suffering from DCM.

## Author contributions

Concept/design: TA; Data analysis/interpretation: TH, IT, TA; Drafting article: TH, IT, TA; Critical revision and approval of article: TA. All authors reviewed the manuscript and approved the final version of the manuscript.

## Conflicts of interest statement

The authors declare that there are no conflicts of interest. Though T.M. is an editorial board member of the JMJ, we declare that he was not involved in the peer review or decision-making process for this paper.

## Supplementary Material

Figure S1 The sequence of the TCAP gene
The bold letters depict Exon 1 and 2 (yellow-highlighted). The guiding RNA was indicated as the blue-highlighted sequence and the site deleted by CRISPR-Cas9 was underlined.

Figure S2 The DNA sequence analysis of the WT (upper) and the gene-edited (lower) iPSCs
The sequencing was analyzed using ICE Analysis (SYNTHEGO; https://ice.editco.bio/#/). The black line shows the guide RNA target sequence, and the red dotted line shows the PAM sequence. The deletion site corresponds to the 14 nucleotides (chr17:39665460_39665473).

Figure S3 The DNA sequence of the TCAP WT and KD
Exon1 and 2 were yellow-highlighted and the guiding RNA was indicated as the blue-highlighted sequence. The stop codon (TGA) was green-highlighted.

Figure S4 The amino acids (aa) sequence of the TCAP WT and K
A truncated polypeptide of 63 aa was predicted in TCAP KD compared with full length telethonin protein of 167 aa.

Figure S5 The result of the SpliceAI simulation
The SpliceAI (BROAD institute; https://spliceailookup.broadinstitute.org/) simulation showed a high probability of loss of the splice donor site.
