# Supplementary figures and images for: Knockdown of Telethonin Reduces Contractions and Provokes Aberrant Ca2+-waves in Human iPS Cell-induced Cardiomyocytes
Source: Juntendo Med J. 2025 Jun 20;71(4):247–55. doi: 10.14789/ejmj.JMJ24-0025-OA (PMC12441175; doi:10.14789/ejmj.JMJ24-0025-OA)

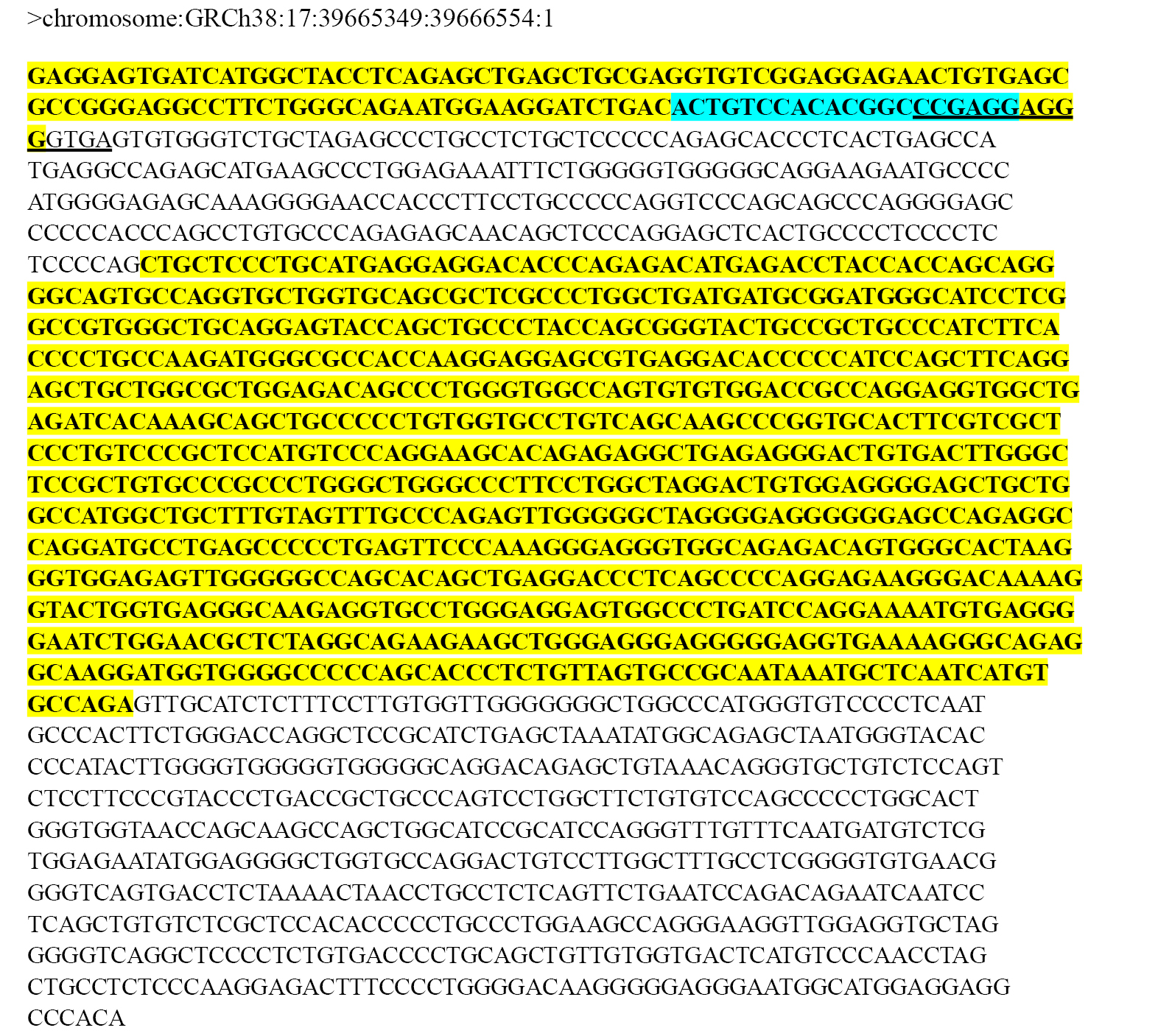

Supplement: Figure S1 — The sequence of the TCAP gene The bold letters depict Exon 1 and 2 (yellow-highlighted). The guiding RNA was indicated as the blue-highlighted sequence and the site deleted by CRISPR-Cas9 was underlined. [file 2759-7504-71-4-0247-s001.jpg]

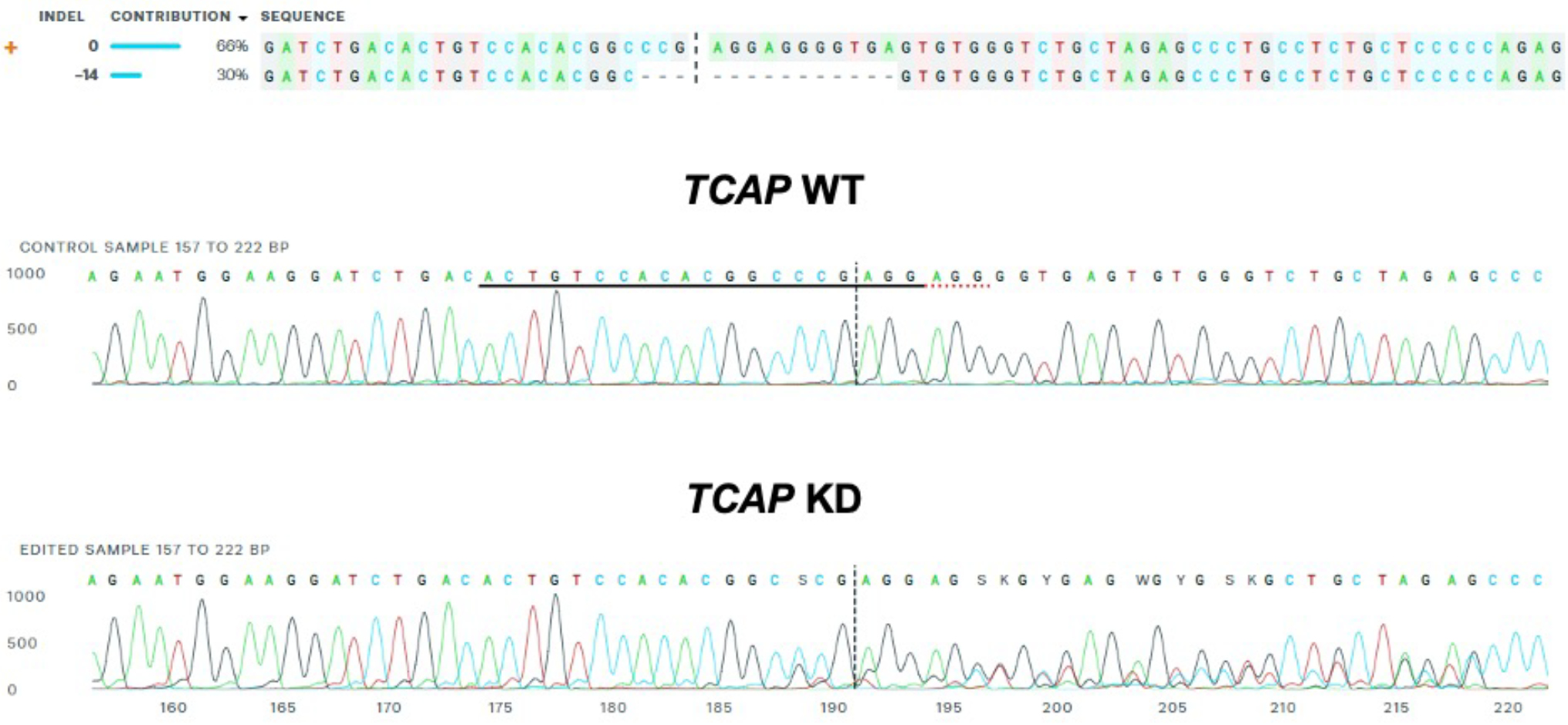

Supplement: Figure S2 — The DNA sequence analysis of the WT (upper) and the gene-edited (lower) iPSCs The sequencing was analyzed using ICE Analysis (SYNTHEGO; https://ice.editco.bio/#/). The black line shows the guide RNA target sequence, and the red dotted line shows the PAM sequence. The deletion site corresponds to the 14 nucleotides (chr17:39665460_39665473). [file 2759-7504-71-4-0247-s002.jpg]

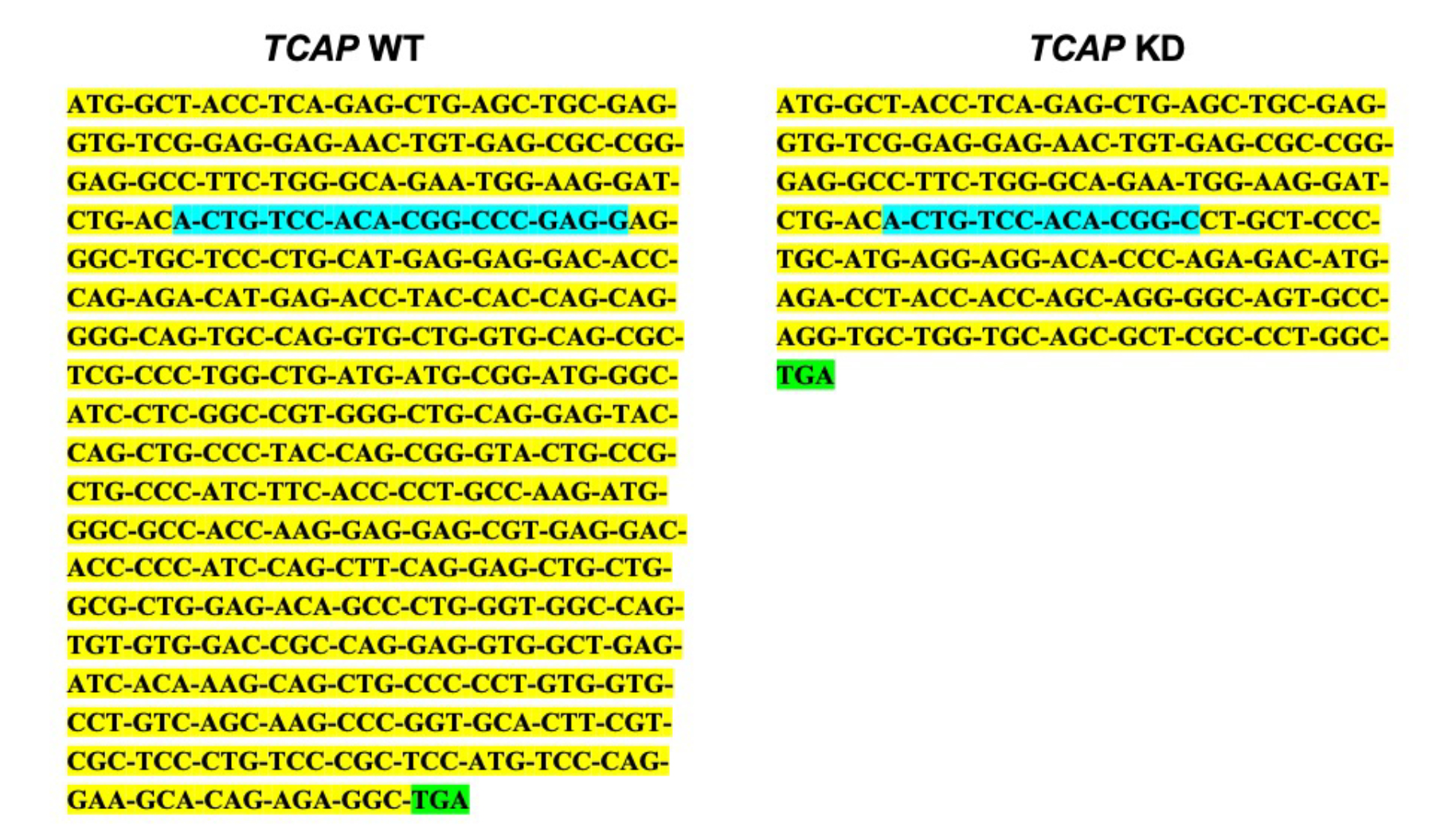

Supplement: Figure S3 — The DNA sequence of the TCAP WT and KD Exon1 and 2 were yellow-highlighted and the guiding RNA was indicated as the blue-highlighted sequence. The stop codon (TGA) was green-highlighted. [file 2759-7504-71-4-0247-s003.jpg]

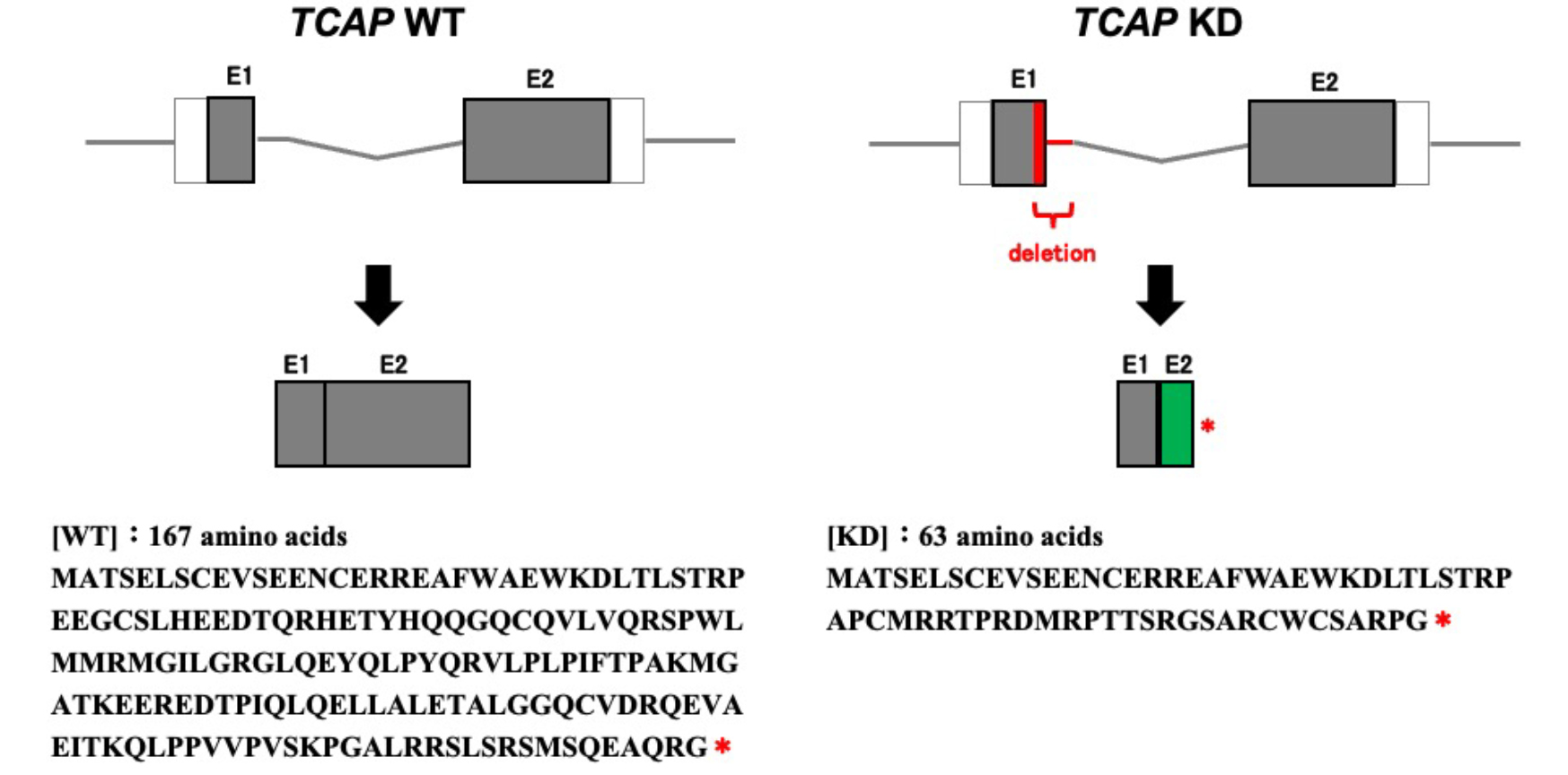

Supplement: Figure S4 — The amino acids (aa) sequence of the TCAP WT and K A truncated polypeptide of 63 aa was predicted in TCAP KD compared with full length telethonin protein of 167 aa. [file 2759-7504-71-4-0247-s004.jpg]

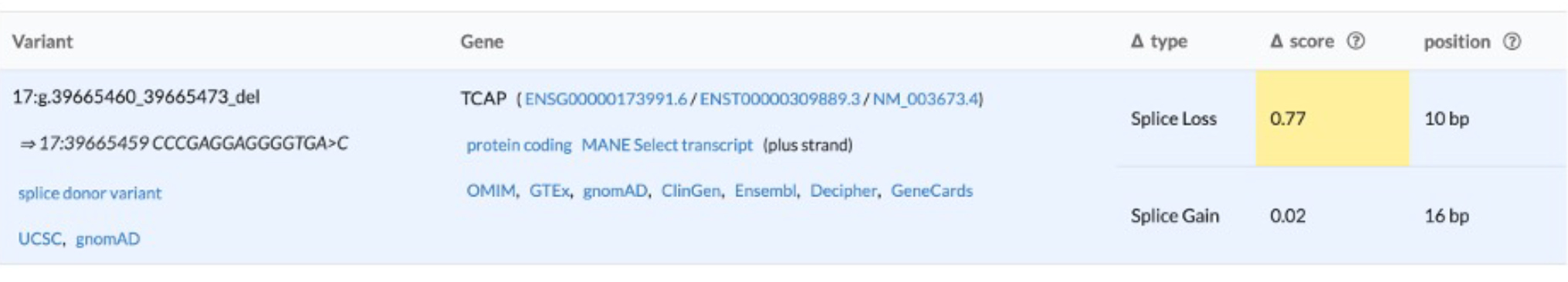

Supplement: Figure S5 — The result of the SpliceAI simulation The SpliceAI (BROAD institute; https://spliceailookup.broadinstitute.org/) simulation showed a high probability of loss of the splice donor site. [file 2759-7504-71-4-0247-s005.jpg]
